# Vaccination Reduces Fecal Shedding and Improves Carcass Quality in Pigs with Subclinical *Lawsonia intracellularis* Infections

**DOI:** 10.3390/vaccines13070728

**Published:** 2025-07-04

**Authors:** Rubén Del Pozo Sacristán, Hanny Swam, Stephan von Berg, Amy Elizabeth Taylor

**Affiliations:** 1 MSD Animal Health, Milton Keynes MK7 7AJ, UK; 2 MSD Animal Health, 5831 Boxmeer, The Netherlands; hanny.swam@merck.com; 3 MSD Animal Health, 85716 Munich, Germany; stephan.von.berg@msd.de; 4 National Pig Centre, Faculty of Biological Sciences, University of Leeds, Leeds LS2 9JT, UK; a.e.taylor@leeds.ac.uk

**Keywords:** *Lawsonia intracellularis*, subclinical infections, vaccination, fecal shedding, growth, tail biting

## Abstract

**Background/Objectives**: *Lawsonia intracellularis* is a bacterium that causes Proliferative Enteropathy, an enteric infection characterized mainly by diarrhea and growth retardation, leading to important economic losses. Acute and chronic infections are easily diagnosed, and their control by vaccination has been proven efficacious. However, subclinical infections, despite being very prevalent, often remain underdiagnosed and uncontrolled in practice. Scarce research is available on the control of subclinical infections by vaccination, and the benefit in these scenarios remains to be elucidated. Two field trials were carried out to (1) determine the association between the growth and fecal shedding of *L. intracellularis* in unvaccinated and intramuscularly vaccinated pigs in a farm with subclinical infection and (2) assess the impact of intradermal vaccination against *L. intracellularis* on clinical performance and carcass quality in a herd with subclinical infection. **Methods**: A pig herd with subclinical infection was selected. Pigs were vaccinated intramuscularly (study 1) or intradermally (study 2) at weaning. Fecal shedding, performance, clinical parameters, and carcass quality were investigated. **Results**: Growth was negatively associated with the fecal load of *L. intracellularis* in non-vaccinated pigs, whereas in vaccinated pigs, growth performance was not impacted by fecal load (study 1). Vaccinated pigs presented a significantly lower fecal load, lower prevalence of tail biting (31.7%) compared with controls (54.2%), less back fat, and a greater Lean Meat percentage (study 2). **Conclusions**: Vaccination against *L. intracellularis* in a herd with subclinical infection and low fecal bacterial shedding led to a reduction in fecal shedding, a lower prevalence of tail biting, and an improvement in carcass quality.

## 1. Introduction

Porcine Proliferative Enteropathy is an enteric disease caused by *Lawsonia intracellularis,* an intracellular Gram-negative bacterium [1]. This pathogen infects immature intestinal epithelial cells mainly from the ileum, leading to the adenomatous proliferation of an immature intestinal mucosa, which is commonly known as ileitis [2]. This thickening of the intestinal mucosa impairs nutrient absorption, followed by diarrhea and subsequent growth retardation [3].

The clinical presentation of the disease may vary from acute to chronic and subclinical forms. The acute form, known as Proliferative Hemorrhagic Enteropathy, is associated with hemorrhagic diarrhea and sudden death. The impact of the chronic form, or Porcine Intestinal Adenomatosis, originates from the impairment of nutrient absorption, resulting in energy leakage through diarrhea and leading to a loss of growth [2]. In subclinical infections, diarrhea is uncommonly seen; however, the gut mucosa is colonized by a moderate number of *L. intracellularis* bacteria, and intestinal lesions are already extensive [4,5,6], indicating that energy leakage through the loss of amino acids and proteins occurs [7]. Winkelman [8] defined subclinical ileitis as a confirmed laboratory diagnosis through the detection of the bacterium by PCR or antibodies, along with the presence of macro- and/or microscopic lesions, but without any ileitis-associated clinical signs or mortality. The absence of specific clinical signs and ileitis-associated mortality makes this form invisible to the eyes of the animal caretaker or the diagnostician, particularly when precise and reliable growth recordings are unavailable [9].

Numerous surveys have shown high herd prevalence in farms infected with this bacterium in Europe (70–100%) [6], the US (75–78%) [10], Brazil (73–98%) [11,12], and Asia (77–100%) [13,14,15,16,17]. Despite this high prevalence of *L. intracellularis*-positive farms worldwide, the number of farms diagnosed in practice with either acute or chronic infections remain limited, whereas a significant proportion of subclinically infected farms continues to be underdiagnosed [4,18,19]. In addition, the use of antimicrobials hinders the detection of these subclinical infections. Fortunately, several studies have given visibility to the economic relevance of *L. intracellularis* subclinical infections and their negative impact on growth [19,20,21]. Interestingly, all these studies have quantified the fecal bacterial load of infected pigs by using a real-time polymerase chain reaction (qPCR) [22,23] and then have demonstrated a positive association between this fecal load and both microscopic lesions and growth retardation caused by *L. intracellularis* infections. According to Collins [5] and Johanssen [24], fecal shedding needs to be more than 10^6^ GE/g feces and 10^7^ GE/g feces before having an impact on growth. Similarly, Pedersen [25] found that the lower limit for detection in pigs with histological lesions was 4.8 Log10 GE/g feces.

Current uncertainties regarding raw materials supply, pork prices, and global trade redirect the focus of the swine industry to improving feed efficiency and sustainability. Consequently, the optimization of growth and performance using less feed is of paramount importance for the economic viability of every pig herd, particularly those subclinically infected. This strategy is included in the road map of every control program for *L. intracellularis* infections, and different tools are available to achieve this goal, including antimicrobial treatment, vaccination, and the optimization of management, hygiene, and biosecurity [2]. In the current context of antimicrobial reduction, vaccination is gaining relevance in this control approach. Vaccination, both intramuscular and intradermal, has been proven to be an excellent tool to reduce clinical signs, bacterial shedding in feces, and performance losses in clinically affected farms [26,27,28,29,30,31]. However, it is still unknown whether vaccination could positively impact performance in this large population of farms with subclinical infection and low fecal bacterial shedding. Our study aims to address this knowledge gap, frequently acknowledged in practice by pig producers and practitioners.

The overall aim of this study was to assess the clinical, growth, and carcass performance of intramuscular or intradermal vaccination against *L. intracellularis* in a herd with subclinical infection and low fecal bacterial shedding. Two studies were carried out:

1. Field trial 1 was performed to elucidate whether there was an association between the growth and fecal shedding of *L. intracellularis* in unvaccinated and intramuscularly vaccinated pigs in a farm with subclinical infection.
2. Field trial 2 was performed to assess the impact of intradermal vaccination against *L. intracellularis* on clinical performance and carcass quality in a herd with subclinical infection.

## 2. Materials and Methods

### 2.1. Herd Selection

A herd with a history of subclinical ileitis and a low bacterial load in feces was selected for both studies. In our study, subclinical ileitis was defined (adapted from Winkelman) [8] by the absence of ileitis-associated clinical signs and/or mortality, as well as by the detection of the bacterium by qPCR. A low bacterial fecal load was considered when the quantification of genomic copies by RT-PCR (Bactoreal^®^, Kit *Lawsonia intracellularis*, Ingenetix GmbH, Wien, Austria) [32] did not exceed 4 Log10 GE copies/µL. This cut-off was established based on several scientific references [5,24,25] and guided by practical experience in the field (personal communication).

Before the start of the study, cross-sectional fecal sampling was performed, and qPCR testing was performed for the quantification of *L. intracellularis*. Pigs at 6, 9, 12, 15, 18, and 21 weeks of age were screened. For each age group, a total of 15 fresh fecal samples were collected from the floor of different pens and pooled by 5. Each pool included at least 25 g of feces and was analyzed by PCR for the detection of *L. intracellularis, Brachyspira hyodysenteriae* (Kylt® BHP Triplex, SAN Group Biotech Germany GmbH, Höltinghausen, Germany), *Brachyspira pilosicoli* (Kylt® BHP Triplex, SAN Group Biotech Germany GmbH, Höltinghausen, Germany), and *Salmonella enterica* Tiphimurium (Kylt^®^ Salm, SAN Group Biotech Germany GmbH, Höltinghausen, Germany). Only the samples from 9-, 12-, and 15-week-old pigs tested positive for *L. intracellularis* by PCR. The bacterial loads for those positive age groups were in the ranges 0–2.9, 1.5–2.7, and 2.8–3.8 Log10 copies/µL, respectively. All samples tested negative by PCR for *B. hyodysenteriae* and *Salmonella* Typhimurium, whereas *B. pilosicoli* was only detected at 18 weeks of age.

The herd was a farrow-to-finish farm housing 400 sows, with a commercial health status, working in a 3-week-batch farrowing system and weaning at 28 days of age. Prior to weaning, piglets were routinely vaccinated against PCV2 and *M. hyopneumoniae* (Porcilis^®^ PCV M Hyo, MSD Animal Health, Rahway, NJ, USA). No routine antimicrobial medication was administered to the piglets during the nursery period (from weaning to 11 weeks of age), nor during the fattening period (from 12 weeks to slaughter). Only zinc oxide was administered in the first two weeks post-weaning to control post-weaning diarrhea. Prior to the initiation of the study, ethical approval was obtained from University of Leeds (20211404ATKA).

### 2.2. Field Trial 1

A randomized, controlled, blind, side-by-side trial was performed. In total, 240 pigs were included in the study and individually tagged within 24 h of birth. On the day of vaccination (28 days of age; day 0), all piglets were weighed and ranked within litter from the heaviest to the lightest; then, every alternate pig was allocated to either the Vaccination [V; n = 120; vaccinated with Porcilis^®^ Lawsonia (lyophilized pellet) (MSD Animal Health, Rahway, NJ, USA) mixed with Porcilis^®^ PCV M Hyo (2 mL) (MSD Animal Health, Rahway, NJ, USA)] or Control [C; n = 120; Porcilis^®^ PCV M Hyo (2 mL)] group. Porcilis^®^ Lawsonia consists of both a freeze-dried antigen fraction and a solvent fraction (Emunade^®^). The freeze-dried antigen could be reconstituted prior to use in the solvent. Alternatively, it could be also reconstituted in Porcilis PCV^®^ M Hyo, which is based on the same adjuvant [26]. Therefore, both vaccines given to Group V could be administered in an associated mixed use. No sham-vaccinated group was included due to ethical reasons. Immediately following vaccination, pigs were weaned into slatted pens (20 pigs per pen) according to the vaccination type, matched as far as possible for litter origin, sex, and live weight. Pigs were fed standard commercial diets following weaning and through to slaughter. These diets provided sufficient nutrient levels to satisfy minimum requirements and normal growth performance. Pigs had *ad libitum* access to feed and water at all times.

A longitudinal sampling was carried out by collecting feces on days 0, 21, 42, 63, 84, and 105 from the same individual pig at each time point. Fecal samples (n = 30/group) were taken directly from the anus at defecation. The fecal bacterial load of *L. intracellularis* was assessed by qPCR (Bactoreal^®^, Kit *Lawsonia intracellularis*, Ingenetix GmbH, Wien, Austria), and the Area Under the Curve (AUC) was calculated for each group. The qPCR assay used is a real-time PCR for the detection of *L. intracellularis* DNA, targeting the 16S rRNA gene of *L. intracellularis* [32]. The analytical sensitivity is 10 target copies/PCR reaction. The analytical specificity allows for the detection of so-far-known *L. intracellularis* strains, with no cross-reactions observed to other bacterial isolates according to manufacturer specifications.

Growth performance was investigated using precision farming technology. The daily weights and daily feed intake of the individual pigs were determined throughout the trial by using pig performance testing technology. From weaning to approximately 35 kg, pigs were housed in a specific weaner grower accommodation with the Pig Insight Asserva System (Asserva, Lamballe-Armor, France). These pens were 3.5 m × 2.5 m and housed 20 pigs each. At 35 kg, the pigs were moved into finisher accommodation with the Pig Performance Nedap ProSense system (Nedap livestock management, Groenlo, The Netherlands). Each nursery pen of 20 was split into two fattening pens of 10. The pens in the finisher accommodation were 2.6 m × 3.5 m and housed 10 pigs each. All automated or mechanical equipment was inspected at least once per day. Pigs were sent to slaughter at approximately 110 kg live weight. Average Daily Gain (ADG), Average Daily Feed Intake (ADI), and Feed Conversion Ratio (FCR) were calculated using the data generated by the aforementioned technology. The association between bacterial load and ADG, ADI, and FCR for the 7 days prior to the peak of bacterial shedding was determined.

Pig health was assessed and recorded daily. A record was kept of any veterinary interventions. Mortality was documented during the whole trial and post-mortem examinations were carried out for those casualties at a certified diagnostic lab.

### 2.3. Field Trial 2

A randomized, controlled, blind, side-by-side trial was performed. In total, 240 pigs were included in the study and individually tagged within 24 h of birth. On the day of vaccination (28 days of age; day 0) all piglets were weighed and ranked within litter from the heaviest to the lightest; then, every alternate pig was allocated to either the Vaccination group [V; n = 120; vaccinated with Porcilis^®^ Lawsonia ID (lyophilized pellet) (MSD Animal Health, Rahway, NJ, USA) mixed with Porcilis^®^ PCV ID (0.2 mL) (MSD Animal Health, Rahway, NJ, USA) and administered concurrently with Porcilis^®^ M Hyo ID Once (0.2 mL) using and IDAL^®^ Twin (Henke Sass Wolf GmbH, Tuttlingen, Germany)] or the Control group [C; n = 120; Porcilis^®^ PCV ID (0.2 mL) and Porcilis^®^ M Hyo ID Once (0.2 mL) administered concurrently using and IDAL^®^ Twin]. No sham-vaccinated group was included due to ethical reasons. Immediately following vaccination, pigs were weaned following the same protocol as described for study 1.

A longitudinal sampling was performed by collecting feces on days 0, 21, 42, 63, 84, 105, and 126 from the same individual pig at each time point. Fecal samples (n = 30/group) were taken directly from the anus at defecation. The fecal bacterial load of *L. intracellularis* was assessed by a qPCR test (Bactoreal^®^, Kit *Lawsonia intracellularis*, Ingenetix GmbH, Wien, Austria), and the AUC was calculated for each group.

Ileitis-associated mortality %, treatment incidences, scour incidences, and tail biting % were registered. At slaughter, carcass quality was assessed by recording carcass weight, back fat level (at P2 level; cm), and Lean Meat % (LM%).

### 2.4. Statistical Analysis

A Kruskal–Wallis test was run to evaluate bacterial shedding and carcass characteristics. Proportions of mortality, antibiotic treatment, and tail bitten incidences in each group were assessed by Chi-square analysis.

Additionally, in study 1, the performance data were analyzed using a mixed linear model procedure (IBM SPSS Statistics 26) with pig as the experimental unit. Treatment was included as a fixed effect. A random effect of pen nested within treatment was also included. Normality was assessed using the Shapiro–Wilk test. Any data that were not normally distributed were subject to a log10 transformation prior to analysis. Transformed data were back-transformed prior to inclusion in the presented results. A linear regression was used to assess the association between the number of *L. intracellularis* bacteria in feces and ADG, ADI, and FCR for the 7 days prior to the pigs testing positive. A sample size of 110 pigs per group was estimated to detect a 6% difference in ADG between treatment groups with a power of 90%. Then, 10 pigs were added to top each group up to 120 pigs to cover any losses.

## 3. Results

### 3.1. Field Trial 1

#### 3.1.1. Fecal Bacterial Load

**Figure 1:**
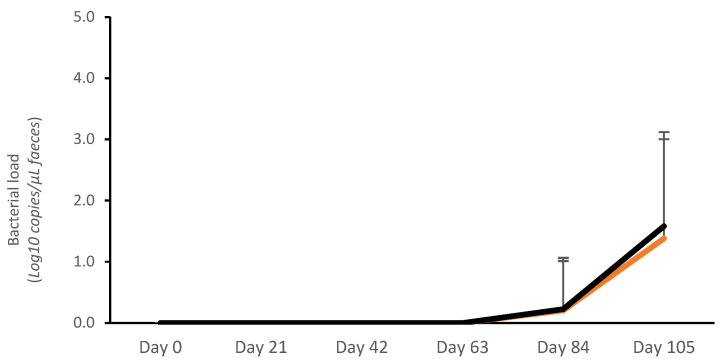
Average *Lawsonia intracellularis* shedding in feces in vaccinated pigs (orange) and control pigs (black).

No bacterial shedding was detected from day 0 to day 63. The average bacterial load (Log10 copies/μL) was very low in both vaccinated and control pigs on day 84 (V: 0.19 ± 0.13; C: 0.20 ± 0.14; *p* > 0.05) and day 105 (V: 1.17 ± 0.27; C: 1.48 ± 0.27; *p* > 0.05). The average AUC for fecal bacterial load on days 0–105 was numerically lower for vaccinates (14.96 ± 20.64 Log10 copies/μL) than for controls (17.53 ± 19.85 Log10 copies/μL) (*p* > 0.05) (Figure 1). There was also a numerical reduction in the proportion of pigs that tested positive for *L. intracellularis* by qPCR in the vaccinated group (46.7%; 14/30) compared with the controls (63.3%; 19/30) (*p* > 0.05).

#### 3.1.2. Performance

Live weight was similar for each treatment group on day 0 (V: 8.85 kg; C: 8.86 kg; *p* > 0.05). Subsequently, there was a tendency for vaccinated pigs to be heavier from day 7 to day 105 (*p* < 0.1). This average weekly live weight was consistently higher in vaccinated pigs than in the controls, being significant only on day 77 (2.2 ± 0.77 kg). Overall, the treatment groups performed similarly (Table S1).

#### 3.1.3. Association Between Fecal Load and ADG, ADI, and FCR

The peak of bacterial load was confirmed on day 105 (Figure 1), and this was concurrent with the largest proportion of PCR-positive samples. Therefore, the association between bacterial load and ADG, ADI, and FCR was calculated for the 7 days prior to this peak of infection (day 99–day 105). During this period, an association between the number of *L. intracellularis* bacteria in feces and ADG, ADI, and FCR was described (Table 1).

A significant negative correlation between the ADG of control pigs and Log10 bacteria per gram of feces was described (R2 = −0.193; *p* < 0.05); the higher the bacterial load in feces, the lower the rate of gain. This negative correlation was not observed in vaccinated pigs (Figure S1).

There was a negative association between the ADI of control pigs and Log10 bacteria per gram of feces (R2 = −0.111; *p* < 0.1); the higher the bacterial load in feces, the lower feed intake. This negative correlation was not observed in vaccinated pigs (Figure S2).

A positive correlation between the FCR of control pigs and Log10 bacteria per gram of feces was detected (R2 = 0.136; *p* < 0.05); the higher the bacterial load in feces, the higher the FCR. No significant correlations were observed between FCR and Log10 bacteria per gram of feces in vaccinated pigs (Figure S3).

#### 3.1.4. Clinical Parameters

Clinical signs compatible with *L. intracellularis* infection were not recorded. Ileitis-associated mortality was not reported. There was no difference in the number of pigs treated with antibiotics or that died between treatments throughout the trial (Table 2). Tail biting was documented in both groups, and the proportion of pigs affected was not significantly different between the groups.

### 3.2. Field Trial 2

#### 3.2.1. Fecal Bacterial Load

**Figure 2:**
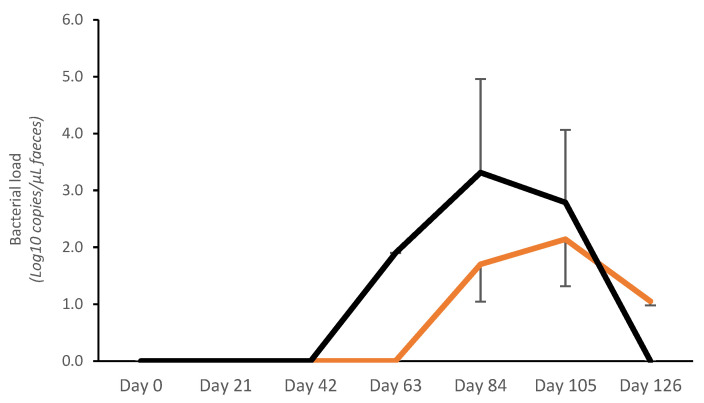
Average *Lawsonia intracellularis* shedding in feces in vaccinated pigs (orange) and control pigs (black). Average Area Under the Curve (bacterial shedding Log10 copies/μL) from day 0 to day 126 was significantly lower for vaccinates (20.72 ± 25.93) compared with controls (40.23 ± 39.10) (*p* < 0.05).

No bacterial shedding was detected before day 63 in controls, whereas in vaccinated pigs, fecal shedding started later, on day 84 (Figure 2). A significantly lower bacterial load (Log10 copies/μL) was detected on day 84 in vaccinated pigs (V: 1.70 ± 0.66; C: 3.31 ± 1.65; *p* < 0.05). The average AUC (bacterial shedding Log10 copies/μL) from day 0 to day 126 was significantly lower in vaccinates (20.72 ± 25.93) compared with the controls (40.23 ± 39.10) (*p* < 0.05) (Figure 2).

#### 3.2.2. Clinical Parameters

**Figure 3:**
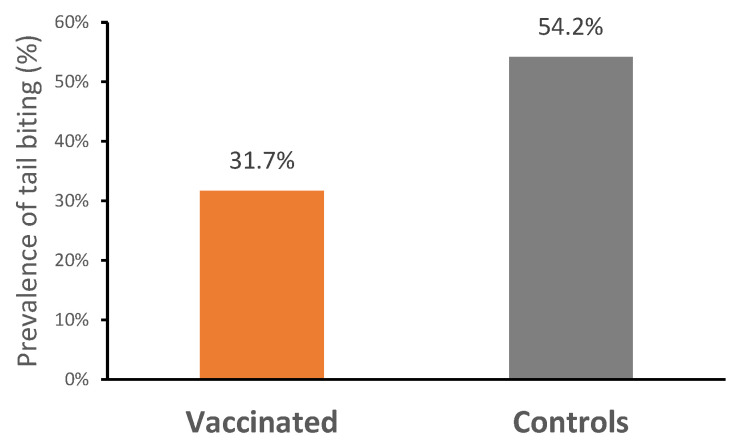
Prevalence of tail biting in vaccinated and control pigs (*p* < 0.05).

The mortality rate was very low across the entire study (V: 0%; C: 0.8%; *p* > 0.05), and no ileitis-associated mortality was recorded. During the first 21 days of the study, a total of 14 piglets were removed from the trial (V: 10; C: 4), due to a failure to adapt to the electronic feeders, which led to low feed intake and poor growth. There was no difference in the number of pigs treated with antibiotics between treatments throughout the trial (Table 3). Treatment incidences related to scour were similar for both groups (V: 11; C: 12; *p* > 0.05). Vaccinated pigs had a significantly lower prevalence of tail biting (31.67%; n = 38) compared with control pigs (54.17%; n = 65) (*p* < 0.05) (Figure 3).

#### 3.2.3. Carcass Quality

Vaccinated pigs had less back fat (10.5 ± 0.14 mm vs. 10.9 ± 0.14 mm; *p* < 0.07) and greater LM% (62.7 ± 0.12% vs. 62.1 ± 0.12%; *p* < 0.05) compared with non-vaccinated pigs (Table 3). No significant differences were seen in the other parameters.

## 4. Discussion

This study was designed to assess the benefits of vaccination against *L. intracellularis* in a herd with subclinical infection and low fecal bacterial shedding. Taking both field trials together, piglet vaccination on a farm with subclinical infection led to lower fecal bacterial shedding, a partial reduction in tail biting, and an improvement in carcass quality. The fecal load of *L. intracellularis* had a negative effect only on the growth of non-vaccinated pigs, whereas growth in vaccinated pigs was not impacted.

While subclinical *L. intracellularis* infections are very common, they remain often underdiagnosed. Consequently, no control strategies are set in place, and the economic performance of those herds is negatively impacted. There is limited research regarding the control of subclinical infections. This study reveals the impact of subclinical infection and sheds light on the use of vaccination to help control any negative impacts, addressing a knowledge gap commonly encountered in the field by pig farmers and practitioners. It also elucidates the relationship between fecal shedding and growth in subclinical cases. In addition, this is the first scientific report showing a partial reduction in tail biting after *L. intracellularis* vaccination.

The preliminary screening carried out before the start of the trials confirmed that the selected farm suffered from a subclinical *L. intracellularis* infection. The absence of both ileitis-like signs and mortality corroborated the suitability of the farm for the study. A low bacterial load in feces was detected before the start of the study. This load was lower than the threshold previously described as the minimum limit for growth retardation [5,21]. However, it was comparable to the cut-off set by Pedersen et al. [4] for the presence of microscopic lesions caused by *L. intracellularis*. It is acknowledged that a high-shedding farm would be more suitable to test vaccine efficacy. However, the study aimed to investigate whether vaccination was still beneficial under these specific conditions.

Fecal bacterial shedding was reduced in vaccinated pigs compared with the controls in trial 2. It is important to note the lack of significance between groups in trial 1. As the overall load was extremely low in trial 1 compared with the threshold for detection of histological lesions (4.8 Log10 GE/g feces) [25], non-significant results are not surprising. However, both the lower AUC and the smaller proportion of PCR-positive pigs in the vaccinated group, even if only numerically different, supported this trend of lower shedding. It is important to note the differences in dynamics and fecal load between the batches of both trials. This is in line with previous reports [31,33] that revealed that the variability in the dynamics and epidemiology of *L. intracellularis*, even between consecutive batches, was a common observation in the field. To avoid underdiagnosing subclinical infections, there is a need for serial longitudinal fecal sampling, even including several batches. This emphasizes the unsuitability of single time fecal sampling for the same purpose. Due to the labor-intensive protocol and high cost of facility usage, our research did not include multiple batches as replicates, which could be a limitation. However, the pre-screening of the herd prior to the start and its history of subclinical ileitis had confirmed the suitability of the herd for the purpose of this study.

Despite a tendency for vaccinated pigs to grow consistently better and faster than controls every week of the study, both treatment groups performed similarly at end of the field trial 1. The lack of significant differences could be explained by the late *L. intracellularis* infection, occurring only four weeks before slaughter, together with the low bacterial shedding in the investigated batch. It is hypothesized that vaccinated pigs did not have enough time between infection and slaughter to express their full potential in growth, as there was not enough time for the unvaccinated, non-protected controls to suffer from the expected retardation in growth. The great variation in infection dynamics between batches likely played a role here. While the pre-screening revealed a low bacterial load (<4 Log10 copies/µL) present between 9 and 15 weeks of age before the start of the study, the batch included in the trial showed a totally different infection pattern, i.e., a late infection around 22 weeks of age characterized by very low fecal shedding (<2 Log10 copies/µL). A future corrective measure when investigating performance in farms with subclinical infections would be the inclusion of several batches as a replicate.

Several attempts have been made to find a relationship between growth rate and the level of antibodies against *L. intracellularis*. However, they have not been successful. In one of those studies [21], a lack of association was shown between reduced growth rate and the level of antibodies against this bacterium. However, the same authors found also that fecal shedding, as measured by qPCR, was a better indicator of growth retardation. More specifically, a high fecal load (>10^6^ copies/g of feces) was a significant risk factor for low growth. Similarly, an Australian study [5] described that the fecal shedding of *L. intracellularis* correlated positively with the severity of histopathological lesions of Proliferative Enteropathy and negatively with ADG. In this experimental challenge trial, pigs shedding from 10^7^ to 10^8^ copies/g experienced a large growth retardation (ADG: −131 g/day) compared with non-challenged pigs, whereas when lower shedding was detected (10^6^–10^7^), a smaller impact on ADG was reported (ADG: −15 g/day). These observations confirmed that antibodies reflect the immune response to infection, whereas fecal bacterial load mirrors the infection level in the intestine. Another Danish longitudinal field study also reported a negative correlation between bacterial load in feces and ADG in naturally infected but non-vaccinated pigs [20].

While the aforementioned studies described this association in animals clinically affected with high bacterial shedding, others have also reported a similar association in pigs with subclinical infections [19]. These authors were able to experimentally induce the transition from clinical to subclinical infection and impair performance by inoculating different doses of *L. intracellularis,* demonstrating a dose-dependent response. Growth retardation (ADG) was the most sensitive indicator of disease, and it revealed the impact of subclinical infections. Lower ADG (−37%; −63 g/day) and feed efficiency (−27%) were recorded at the lowest challenge dose (3.2 × 10^4^ organisms). In the absence of clinical signs of disease, pigs from the lowest challenge group presented sporadic shedding as well as macro- and microscopic lesions. The severity of gross and histological lesions was also dose-dependent.

Abundant research has been carried out under both experimental and field conditions, including both clinically and subclinically infected pigs. However, it is still unknown whether vaccination could have an influence on this association in farms with subclinical infection and low fecal bacterial shedding. One of the aims of our study was to answer this. In our case, an increasing number of *L. intracellularis* log10 bacteria/g of feces was significantly associated with decreasing ADG, but this observation was only confirmed in non-vaccinated pigs: the higher the fecal bacterial load, the lower the rate of weight gain. Conversely, the growth of vaccinated pigs was not negatively affected by the load of *L. intracellularis* in feces. This supports the field observations suggesting that vaccination protects against the disease even if the bacterium is still detected by PCR during routine diagnosis in vaccinated pigs. Now it also indicates that growth is not impacted. This is in line with the benefits shown by both intramuscular and intradermal vaccination on the reduction in *L. intracellularis* shedding in both intestinal mucosa and feces [26,28]. However, it was also shown that the bacterium could still be found, and total elimination was not achieved.

Our findings confirm the impact of these subclinical infections on growth and corroborate the observations by Paradis et al. [19] but slightly conflict with the ones by Pedersen et al. [20]. In their study, no differences in ADG were reported between negative pigs and pigs with low numbers of bacteria in feces [20]. In order to reduce bias, our farm selection followed similar standards to those included in the Danish study. Although our study herd was not an SPF farm, the preliminary screening confirmed the absence of most enteric diseases that may have interfered with *L. intracellularis*. The correlation between growth and shedding was performed taking into consideration only the period of seven days prior to the collection of the samples, which was the same period used in the association modeled by Pedersen et al. [20]. The assay used to quantify bacterial fecal load differs between studies. To the authors’ knowledge, there is only one study comparing different PCR assays for this bacterium [34]. In the research study, two RT-PCRs were included, and both assays presented similar sensitivity and specificity in detecting *L. intracellularis* in fecal samples. The first assay [32], which is the same commercial assay used in our study, presented 97% sensitivity and 85% specificity, as well as 100% analytical specificity. In addition, it was reported that the lower detection limit of the commercial kit was 4 × 10^4^ GE/g of feces. However, the lower quantification limit, robustness, and precision of the test were not reported. For the second assay, the diagnostic performance of the test was reported concisely [22], showing similarities to the previous one (sensitivity of 97%; specificity of 34%; analytical specificity of 100%; lower limit of quantification of 10^1^ GE/µL). In view of this similar diagnostic performance, it was decided to use the commercial kit [32], which had high availability across Europe and is used commercially to support diagnosis in practice.

Regarding ADI and FCR, our study reported a similar trend to the association described for ADG. In non-vaccinated pigs, the higher the bacterial load, the lower the feed intake and the lower the feed efficiency reported. This was not the case for vaccinated pigs. It is assumed that non-vaccinated, unprotected pigs could have suffered from the disease to a higher extent compared with vaccinated ones, developing more severe intestinal lesions, having a lower appetite, and facing suboptimal nutrient absorption which eventually led to lower performance. Unfortunately, this assumption could not be confirmed as an ileum lesion scoring was not carried out. In a previous study [4], no correlation was found between fecal load and gross lesions. However, these authors demonstrated that an increase in histopathology and immunohistochemistry scores was positively correlated with *L. intracellularis* shedding. The lack of microscopic investigation of intestinal tissue is a limitation of our study. Assessing histological lesions would have been beneficial in explaining the growth differences between groups and potentially linking those differences to nutrient absorption at the gut level. However, it is important to note that interpreting histopathology results in practice is challenging, especially when this is performed only at slaughter time rather than through serial investigations, as lesions may heal by the time of slaughter [35].

The use of precision farming technology for the recording and investigation of growth performance was a great asset for the research. It facilitated the detection of patterns and small deviations, while routine weighing at entry and before slaughter would have missed this information. Moreover, it optimized the detection of piglets with lower feed intake and accelerated their exclusion from the study, avoiding important bias. Both precision farming technologies used for live weight recording (the Pig Insight Asserva System and the Pig Performance Nedap ProSense system) presented the advantage of recording it multiple times per pig per day, increasing accuracy and leading to a data set with multiple dependent measurements. Therefore, a Generalized Mixed Linear Model, which can account for the non-independence of the data by adding random effects, was used.

As expected in a farm with subclinical infection, no ileitis-associated clinical signs nor mortality was reported in either group during the study. Similarly, antimicrobial incidences and overall mortality were low and equal between groups. Intramuscular vaccination against *L. intracellularis* has been reported as an alternative tool to reduce antimicrobial use in cases of both acute and chronic ileitis [36,37]. Further research is needed to elucidate if antimicrobial consumption can be also reduced in farms with subclinical infections, where prophylactic use is often still implemented, especially outside of Europe.

Tail biting was reported in both studies. Surprisingly, only vaccinated pigs from trial 2 presented a significantly lower prevalence compared with the controls. To the authors’ knowledge, this is the first scientific report of partial reduction in tail biting after vaccination against *L. intracellularis*. It is unknown why no differences were reported between groups in trial 1. The low bacterial load in feces in trial 1, as well as the use of different routes of administration in each study, might have influenced this outcome, but this study design does not allow us to demonstrate causality and make such a conclusion. Similarly, from the data reported in this study, it cannot be concluded that vaccination against *L. intracellularis* will have a protective effect against tail biting, as there are some limitations. Firstly, the study did not aim to investigate this question or elucidate the mechanism of action. Therefore, it was not designed in such a way to answer this. Secondly, more thorough research would have been needed, including histology of tail lesions, stress markers, microbiome analysis, and/or a complete risk factor analysis. The multifactorial nature of tail biting is continuously being explored, and all mechanisms behind this behavior are not yet fully understood, making it difficult to predict and control [38]. Risk factors for tail biting may be categorized in those related to the pig or to the environment. Pig-associated factors include but are not limited to genetics, gender, health status, growth, age, and behavior. Environmental factors include feeding, space allowance, climate, flooring, access to manipulable materials, housing, etc. All pig- and environment-related factors described above were similar between vaccinated and non-vaccinated pigs. Pigs were randomized at arrival, housed in the same compartment under uniform environmental conditions across the barn, under similar stock density, receiving the same diet, and having similar access to manipulable materials. Therefore, the absence of any bias that might have influenced the tail biting, growth, or fecal shedding in favor of one group or another could be confirmed. Recent research has provided new evidence of a relationship between the gut microbiome and tail biting behavior, pointing at the gut–brain axis as a potential mechanism to partially explain this phenomenon [39,40,41,42,43]. While studies indicate that *L. intracellularis* can modulate the porcine gut microbiome following either infection [9,44,45] or vaccination [46], no documented association exists between tail biting behavior and this bacterial infection to date. Furthermore, the unexpected findings in our study underscore the need for more extensive research employing an interventional design to investigate behavioral patterns and stress factors associated with gut microbiome dysbiosis and the development of tail lesions.

At slaughter, vaccinated pigs performed better than controls in terms of Lean Meat percentage, presenting less back fat. A similar improvement in carcass quality after *L. intracellularis* intramuscular vaccination has been previously shown [47]. In this study, the authors reported greater carcass weight followed by an increased percentage of lean meat in the ham, loin, and shoulder after intramuscular vaccination. Carcass weight was also increased in another study carried out in Brazil after intramuscular vaccination [48]. In this case, vaccination also improved the integrity of the intestinal wall, leading to an increased yield of casing obtention (meters of casing/intestine) and sausage fill (kg of sausage/meter of casing). Gut inflammation in pigs can significantly impact energy deposition in the carcass. When pigs experience gut inflammation, it often leads to a reduction in feed intake and nutrient absorption, which can negatively affect growth rates and overall carcass quality. Inflammation can trigger the release of pro-inflammatory cytokines, which may divert energy away from muscle synthesis and towards immune responses, leading to increased fat deposition instead of muscle growth [49]. Conversely, low gut inflammation is associated with improved nutrient absorption and metabolic efficiency. When inflammation is minimized, the body can redirect energy towards anabolic processes, including muscle formation. This is facilitated by enhanced biosynthetic pathways, such as protein synthesis and lipid metabolism, which favor muscle growth over fat deposition [50]. Therefore, managing gut health is crucial to optimizing muscle formation and improving carcass quality in pigs. In our study, it was presumed that the reduction in *L. intracellularis* organisms at the gut level in vaccinated pigs led to lower gut inflammation, better gut integrity, and consequently, an improved digestion process and more efficient nutrient absorption. Gut microbiota plays a pivotal role in pig metabolism by leading the fermentation of dietary fiber, the production of Short-Chain Fatty Acids (SCFAs), and the absorption of their metabolites at the intestinal level [51]. SCFAs are instrumental in the protein and fat metabolism of skeletal muscle, affecting muscle fiber formation and intramuscular adipogenesis [51]. Interestingly, they are known to have antimicrobial properties by inhibiting the growth of common enteric pathogenic bacteria and hence supporting the control of intestinal infections [52,53]. However, this study also acknowledges its limitations, particularly the lack of investigation into the specific impacts of vaccination on gut inflammation and gut microbiota composition and how this impacts growth performance and carcass quality. Microbiome analysis, histopathology, metabolic data, and investigations into nutrient absorption will be essential to fully understanding these relationships and develop effective strategies for optimizing energy deposition in the carcass of pigs. By addressing these gaps, we can further enhance the benefits of vaccination and improve the overall efficiency and sustainability of swine production systems.

## 5. Conclusions

In this study, it was shown that vaccination against *L. intracellularis* in a herd with subclinical infection and low fecal bacterial shedding led to a reduction in fecal shedding, a lower prevalence of tail biting, and an improvement in carcass quality. Notably, it was found that growth performance in non-vaccinated pigs was negatively correlated with the fecal load of *L. intracellularis*, whereas vaccinated pigs exhibited no such negative impact, indicating a protective effect of vaccination.

These findings suggest that vaccination can serve as a valuable tool for enhancing farm profitability by improving lean meat yield and mitigating behavioral issues such as tail biting, which can lead to economic losses. By integrating vaccination into herd management practices, producers may not only improve animal welfare but also optimize production efficiency.

Further research is essential to fully elucidating the mechanisms by which vaccination influences tail biting behavior and energy deposition in pig carcasses, paving the way for more effective strategies in swine health management.

## Figures and Tables

**Table 1:** Association (Pearson correlation coefficient; PCC) between the number of *L. intracellularis* bacteria in feces and ADG, ADI, and FCR in control non-vaccinated pigs and pigs vaccinated against *Lawsonia intracellularis*.

	Control Pigs		Vaccinated Pigs	
	PCC	*p*-Value	PCC	*p*-Value
ADG	R2 = −0.193	*p* < 0.05	R2 = 3.831 × 10^−4^	*p* > 0.05
ADI	R2 = −0.111	*p* < 0.1	R2 = 0.008	*p* > 0.05
FCR	R2 = 0.136	*p* < 0.05	R2 = 0.018	*p* > 0.05

PCC: Pearson Correlation Coefficient; ADG: Average Daily Gain; ADI: Average Daily Intake; FCR: Feed Conversion Ratio.

**Table 2:** Clinical parameters recorded in control non-vaccinated pigs and pigs vaccinated against *Lawsonia intracellularis*. No statistical differences were described between groups.

	Control Pigs	Vaccinated Pigs
Mortality %	1.7	0
Individual treatment incidences %	15.8	17.5
Tail biting %	16.7	15.8

**Table 3:** Clinical parameters and carcass quality in vaccinated and control pigs.

	Controls	Vaccinated	Difference
Mortality %	0.8	0	NA
Individual treatment incidences %	32.5	35.8	NA
Tail biting %	54.2 ^A^	31.7 ^B^	−22.5
Carcass weight (kg)	113.7	115.6	NA
Back fat (mm)	10.9	10.5	−0.4
Lean Meat %	62.1 ^A^	62.7 ^B^	+0.6

^A,B^—Different superscripts within the same row represent statistical differences. NA—Not applicable, when no statistical differences were found.

## Data Availability

Please add the corresponding content of this part.

## Supplementary Materials

The following supporting information can be downloaded at: https://www.mdpi.com/article/10.3390/vaccines13070728/s1, Table S1. Overall performance of control non-vaccinated pigs and pigs vaccinated against *Lawsonia intracellularis*. Figure S1. Pearson correlation coefficient between Average Daily Gain (ADG) and bacterial load in feces (qPCR Law; Log10 copies/µL of feces) in control (left) and vaccinated (right) pigs. Figure S2. Pearson correlation coefficient between Average Daily Intake (ADI) and bacterial load in feces (qPCR Law; Log10 copies/µL of feces) in control (left) and vaccinated (right) pigs. Figure S3. Pearson correlation coefficient between Feed Conversion Ratio (FCR) and bacterial load in feces (qPCR Law; Log10 copies/µL of feces) in control (left) and vaccinated (right) pigs.

## Abbreviations

The following abbreviations are used in this manuscript:

qPCR	real-time quantitative polymerase chain reaction
V	Vaccination group
C	Control group
AUC	Area Under the Curve
ADG	Average Daily Gain
ADI	Average Daily Feed Intake
FCR	Feed Conversion Ratio
LM%	Lean Meat percentage
SE	Standard Error
SPF	Specific Pathogen Free
SCFAs	Short-Chain Fatty Acids
