# Supplementary material for: Vaccination Reduces Fecal Shedding and Improves Carcass Quality in Pigs with Subclinical Lawsonia intracellularis Infections
Source: Vaccines (Basel). 2025 Jul 4;13(7):728. doi: 10.3390/vaccines13070728 (PMC12300254; doi:10.3390/vaccines13070728)
Supplement: Supplementary file 1 [file vaccines-13-00728-s001.zip › vaccines-3680333-supplementary.pdf]

Table S1. Overall performance of control non-vaccinated pigs and pigs vaccinated against *Lawsonia intracellularis*.

	Control group	Vaccinated group	SE
**Weaner (day 0 – 21)**			
Weight in (kg)	8.86	8.85	0.564
Weight out (kg)	17.14	17.35	0.188
ADI (g/day)	429	444	13
ADG (g/day)	394	405	9
FCR	1.11	1.12	0.022
**Grower (day 22-56)**			
Weight out (kg)	40.85	41.12	1.229
ADI (g/day)	1183	1198	38
ADG (g/day)	674	679	14
FCR	1.783	1.781	0.026
**Finisher (day 57–slaughter)**			
Weight out (kg)	110.63	110.40	0.753
ADI (g/day)	2675	2766	30
ADG (g/day)	1057	1067	24
FCR	2.44	2.52	0.032
**Weaning (day 0) to slaughter**			
ADI (g/day)	1845	1896	14
ADG (g/day)	853	856	12
FCR	2.18	2.22	0.025

a, b: Different superscripts within the same row show statistical differences.

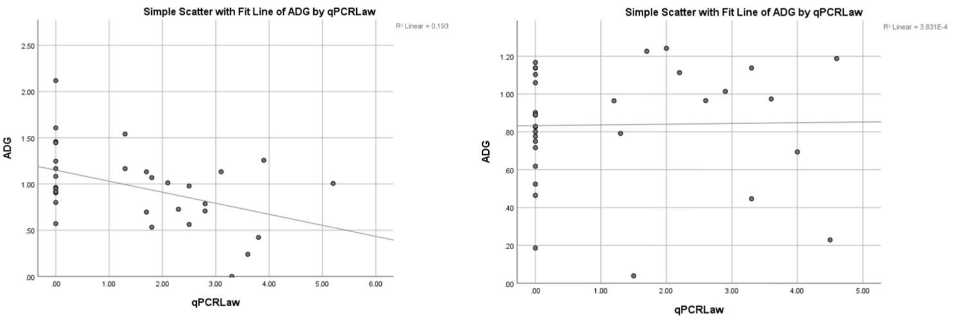

Figure S1. Pearson correlation coefficient between Average Daily Gain (ADG) and bacterial load in feces (qPCR Law; Log10 copies/µL of feces) in control (left) and vaccinated (right) pigs.

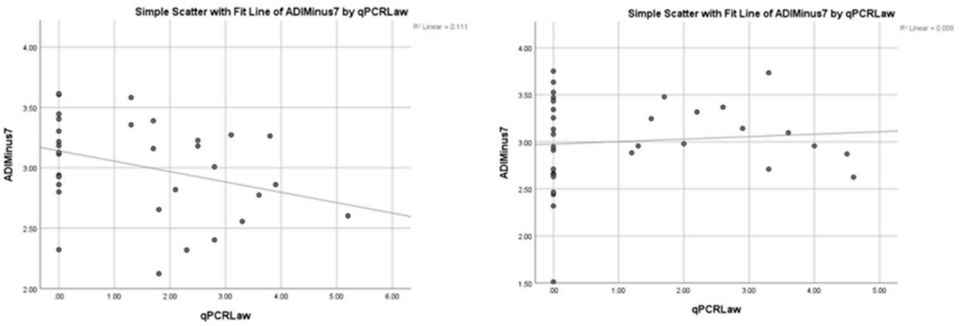

Figure S2. Pearson correlation coefficient between Average Daily Intake (ADI) and bacterial load in feces (qPCR Law; Log10 copies/µL of feces) in control (left) and vaccinated (right) pigs.

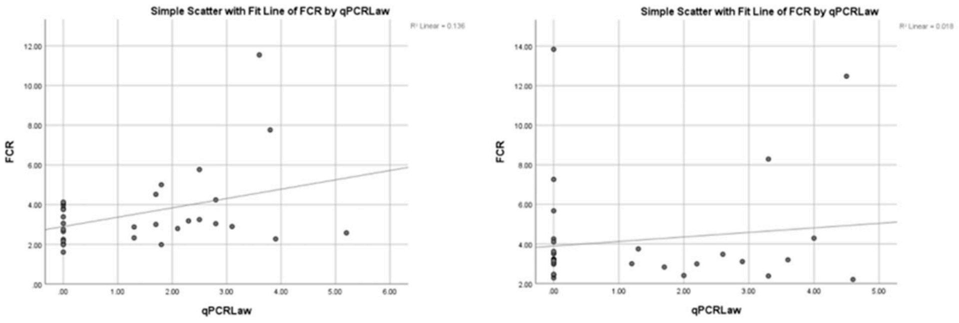

Figure S3. Pearson correlation coefficient between Feed Conversion Ratio (FCR) and bacterial load in feces (qPCR Law; Log10 copies/µL of feces) in control (left) and vaccinated (right) pigs.
